# Integrating ChatGPT into knowledge-retrieval tutorials in undergraduate medical education: a prospective evaluation of higher-order learning and feasibility

**DOI:** 10.1080/10872981.2026.2639203

**Published:** 2026-03-02

**Authors:** Roy Arokiam Daniel, Nivethitha V, Surya BN, Antoinette Daniel, Visalakshi R

**Affiliations:** a Department of Community Medicine, ESIC Medical College and Hospital, KK Nagar, Chennai, Tamil Nadu; b School of Computer Science Engineering (SCOPE), Vellore Institute of Technology (VIT), Chennai, Tamil Nadu; c Department of Community Medicine, Chettinad Hospital and Research Institute, Chettinad Academy of Research and Education, Kelambakkam, Tamil Nadu; d Department of French, School of Social Sciences and Language, Vellore Institute of Technology (VIT), Vellore, Tamil Nadu

**Keywords:** Competency-based medical education, ChatGPT, Generative AI, higher-order thinking, medical education assessment, retrieval practice

## Abstract

**Background:**

Generative artificial intelligence (AI) platforms such as ChatGPT present new opportunities to strengthen competency-based medical education (CBME). While retrieval practice is a proven strategy for enhancing long-term retention, its application to higher-order domains of Bloom’s taxonomy within CBME, particularly when scaffolded by AI, remains underexplored. To our knowledge, this is the first longitudinal CBME study worldwide to evaluate supervised ChatGPT-assisted retrieval practice and its durability over time.

**Methods:**

We conducted a six-month, prospective, non-randomised delayed-intervention study and all 270 third-year MBBS students (including supplementary batch) at a private medical college in South India were invited to participate in a faculty-supervised ChatGPT-assisted retrieval-practice intervention. Participants were allocated into four tutorial clusters; two received the intervention immediately, while the remaining two received it after a delay. The intervention comprised four weekly, two-hour sessions featuring higher-order multiple-choice questions, structured faculty-supervised interactions with ChatGPT, and guided metacognitive reflections. Outcomes assessed included MCQ performance at baseline, immediately post-intervention, and at one- and three-month follow-up, as well as student perceptions. Data analysis employed repeated-measures ANOVA and mixed-effects modelling.

**Results:**

Of 270 students invited, 253 (93.7 %) met inclusion criteria and completed all assessments, achieving 100% follow-up at immediate, one-month, and three-month evaluations. MCQ performance demonstrated significant improvement across time points (F[3,756] = 65.14, *p* < 0.001). On a 20-point higher-order MCQ scale, mean scores increased from 12.8 ± 2.4 at baseline to 15.2 ± 2.1 immediately post-intervention (adjusted gain +2.37, d = 0.62, *p* < 0.001). Gains were sustained at one month (+3.78, d = 0.99) and three months (+3.65, d = 0.95), demonstrating durable higher-order learning retention. Both Lower-achieving and Higher-achieving students improved, though the effect was greater among Lower-achieving students (d = 0.72 vs 0.49). Student feedback revealed high levels of satisfaction (mean 4.25 ± 0.88) and cognitive engagement (4.15 ± 0.92), while clarity of AI interaction received comparatively lower ratings (3.39 ± 1.19).

**Conclusion:**

Supervised ChatGPT-assisted retrieval practice produced sustained improvements in higher-order cognitive performance, with particularly strong benefits for Lower-achieving students. This scalable, standards-aligned model holds promise for advancing CBME globally and warrants further validation through multi-institutional trials incorporating performance-based and clinical outcomes.

## Introduction

Generative artificial intelligence (AI) is reshaping higher education, influencing how students access information, seek feedback, and refine reasoning. In medical education, ChatGPT—a large language model developed by OpenAI—has gained rapid traction for its ability to clarify concepts, simulate clinical reasoning, and respond to complex queries. Within India’s competency-based medical education (CBME) framework, which prioritises integrative thinking and decision-making, many students have already adopted ChatGPT informally. Yet institutional responses remain cautious, citing risks of over-reliance, misinformation, and academic integrity breaches. These concerns, while valid, may obscure AI’s potential when implemented under structured, faculty supervision [1]. In parallel, retrieval practice—deliberate recall of learned material has a robust evidence base for strengthening retention and knowledge transfer. However, its application to Bloom’s higher-order cognitive levels (IV: Analysis, V: Evaluation, VI: Creation) is less explored. These levels underpin clinical competence: diagnosing from incomplete data, weighing evidence to make decisions, and generating novel management plans. Integrating supervised AI into retrieval practice offers a promising pathway to strengthen these skills while maintaining academic rigour [2].

In parallel, decades of cognitive science research have consistently shown that retrieval practice, the active recall of previously learned material is one of the most effective ways to strengthen long-term retention and promote knowledge transfer. Unlike passive review, retrieval engages memory systems more deeply, consolidates knowledge, and supports conceptual restructuring, particularly when paired with delayed feedback and elaborative reasoning. While its value is well documented for factual recall and lower-order cognitive skills (Bloom’s Levels I and II), its application to higher-order competencies such as analysis, evaluation, and creation (Levels IV-VI) remains underexplored. Yet these are precisely the skills that underpin clinical reasoning, population-level decision-making, and integrative problem-solving within the CBME curriculum [3,4].

This study emerged from the convergence of two imperatives in medical education: the growing pedagogical potential of generative AI and the enduring evidence base for retrieval practice. We hypothesised that, when embedded within a structured teaching framework, ChatGPT could function not merely as an information source but as an active cognitive partner prompting metacognitive reflection, encouraging iterative refinement of thinking, and fostering the productive struggle necessary for mastering complex concepts. To our knowledge, this is among the first systematic attempts in medical education to integrate ChatGPT into a faculty-supervised retrieval practice programme designed specifically to target higher-order cognitive skills, implemented within a real-world CBME environment in India. We conducted a prospective, phased, delayed-intervention study with third-year MBBS students in a private medical college in South India, assessing both the educational impact and feasibility of this approach. The primary outcome was improvement in performance on a higher-order summative assessment. Secondary outcomes included retention at one and three months, subgroup differences by baseline academic performance, and students’ perceptions of the intervention through a structured post-intervention survey.

## Materials and methods

This prospective, non-randomised, delayed-intervention study with repeated within-subject assessments was conducted over six months (January-June 2025). A non-randomised design was chosen because true random allocation was not feasible given fixed institutional timetables and administrative constraints governing tutorial group assignments. The delayed-intervention approach allowed internal comparison while ensuring that all students ultimately received the intervention. This study adopted a Hybrid Type II implementation-effectiveness design, which simultaneously evaluates both the educational effectiveness of an intervention and the contextual feasibility of its real-world adoption. In this context, ‘retrieval’ refers to knowledge retrieval; the deliberate, effortful recall of learned information to strengthen higher-order understanding and long-term retention.

### Setting and participants

The study was conducted at a private tertiary-care medical college in South India that follows the CBME curriculum prescribed by the NMC. The institution admits 250 MBBS students annually, with community medicine teaching delivered via a tutorial-based model. Students are pre-assigned to four tutorial clusters (A–D) of approximately 60 students each. Supervised access to GPT-4 was provided via a licensed ChatGPT interface integrated into the institutional Learning Management System (LMS), accessible only in designated seminar rooms over secure Wi-Fi.

An a priori power analysis indicated that detecting an 8-percentage-point improvement in higher-order MCQ scores (SD = 12, *α* = 0.05, power = 80%) required 95 participants by the paired-comparison formula; allowing for 10% attrition gave a target of 110. To enhance generalisability and reduce selection bias, complete enumeration was undertaken by inviting all third-year MBBS students enroled in the 2025–2026 academic cycle (*n* = 270). Although the sanctioned annual intake of the MBBS programme is 250 students, the study cohort included supplementary students from previous batches, resulting in 270 eligible participants. Supplementary students refer to those reappearing for university examinations from earlier batches while progressing with the subsequent cohort. The intervention and follow-up assessments were conducted between August and November 2025. Inclusion criteria were pre-assignment to a tutorial cluster, attendance at the baseline assessment (Form A), written informed consent, and attendance at least three of the four intervention sessions. Exclusion criteria were absence at baseline, attendance at fewer than three sessions, prior exposure to ChatGPT for the same content, or withdrawal of consent. Students were stratified into Lower-achieving students and Higher-achieving subgroups based on their baseline higher-order MCQ performance. Students scoring below the cohort median were categorised as Lower-achieving students, and those scoring at or above the median as Higher-achieving students. This objective, performance-based stratification followed NAAC-aligned formative evaluation practices, ensuring transparency, fairness, and reproducibility while minimising subjective bias.

Socio-economic status (SES) was assessed using the Modified Kuppuswamy Socioeconomic Scale 2025, updated to the current Consumer Price Index [5]. This scale generates a composite score based on the education and occupation of the head of the family and the monthly family income adjusted for inflation, classifying households as Upper (26–29), Upper Middle (16–25), Lower Middle (11–15), Upper Lower (5–10), or Lower (<5). The tool is widely used in Indian community- and hospital-based research to capture socio-economic gradients relevant to educational and health outcomes. In the present study, SES was collected to assess baseline comparability and to serve as a covariate in adjusted analyses, rather than being analysed as a primary outcome.

### Intervention and control conditions

The study followed a phased delayed-intervention rollout. In Week 1, all clusters attended routine tutorials and completed baseline testing (Form A). Clusters A and B then received the intervention during Weeks 2–5 (early-intervention sequence), while clusters C and D continued routine academic activities during this period and subsequently received the intervention during Weeks 3–6 (delayed-intervention sequence). Thus, all clusters ultimately received the intervention, with the distinction lying solely in timing of exposure. All students completed an immediate post-intervention assessment (Form B), followed by one-month (Form C) and three-month (Form D) assessments administered via LMS-embedded Google Forms under supervision. The intervention comprised four weekly, two-hour, in-person sessions led by trained faculty and postgraduate tutors. Each session followed a structured sequence: presentation of five single-best-answer MCQs mapped to Bloom’s Levels IV-VI; independent student responses with justifications entered into ChatGPT; structured interaction with ChatGPT using standardised, faculty-approved prompts (Supplementary Appendix S1); revision of answers based on AI feedback; and a short written reflection capturing metacognitive insights.

During each session, student groups of 8–10 interacted with ChatGPT through a projected institutional account under real-time faculty supervision. Students first entered their justification for each MCQ answer into the chat interface using structured prompts designed to elicit higher-order reasoning rather than factual recall. Faculty tutors observed these exchanges, intervening only when the AI output contained factual inaccuracies, incomplete reasoning, or ambiguous explanations. Interventions typically involved (i) modelling how to challenge an AI response with follow-up queries, (ii) highlighting sources of potential error or bias, and (iii) encouraging students to triangulate AI information with standard references (Park’s Textbook of Preventive and Social Medicine). Supervision was deliberately scaffolded to preserve student autonomy while ensuring epistemic accuracy. Students were encouraged to query ChatGPT iteratively until they could justify their final answer and articulate the reasoning path. The faculty’s role was thus primarily to guide enquiry, maintain academic rigour, and promote metacognitive reflection rather than to provide direct answers (Box 1). During their pre-intervention phase, clusters received identical content and time allocation but without AI access, relying instead on textbooks, notes, and LMS resources, with peer discussion permitted. ChatGPT access was disabled for control groups to prevent contamination. After the study, all students were provided with ChatGPT access, materials, and a faculty-led debriefing. All MCQ items were blueprint-based and psychometrically validated, with parallel forms used across groups to ensure equivalent content coverage and difficulty.

Box 1. Faculty-Supervised ChatGPT-Assisted Retrieval-Practice Workflow.

**Table:** 

Step	Illustrative Example (Condensed from Actual Sessions)
1. Higher-Order MCQ Stimulus (Trigger for Retrieval Practice)	*A 45-year-old man with chronic cough and weight loss is newly diagnosed with pulmonary TB. After two months of treatment, his sputum remains positive for AFB. What is the next best step?* A. Extend intensive phase B. Add levofloxacin C. Repeat DST D. Start MDR-TB regimen E. Continue same regimen
2. Student Reasoning Statement (Initiating AI-Mediated Dialogue)	*‘I selected option C because DST confirms resistance before regimen modification, though I’m uncertain about the correct timing according to NTEP guidelines.’*
3. Structured Prompt to ChatGPT (Eliciting Explanatory Feedback)	*‘Explain which option is correct according to the National TB Elimination Programme. Analyse each option and clarify when DST should be repeated if smear remains positive after 2 months.’*
4. ChatGPT Analytical Response (Summarised Output)	*‘Persistent smear positivity at 2 months suggests possible resistance. NTEP recommends repeating DST before modifying regimen. Hence, option C is correct. Extension or change without DST risks undertreatment or resistance amplification.’*
5. Faculty Supervisory Mediation (Ensuring Epistemic Accuracy)	*Tutor prompts verification using Park’s PSM and NTEP 2023 manual, guides discussion on adherence vs resistance, and models how to frame follow-up questions to evaluate evidence quality.*
6. Learner Reflection and Cognitive Restructuring (Metacognitive Step)	*Student recognises initial bias toward assuming resistance; ChatGPT’s counter example prompted reconsideration of behavioural and programmatic causes of treatment failure.*

Note: Each session used five such MCQs mapped to blueprint competencies and Bloom’s Levels IV–VI. Students entered structured reasoning statements, engaged in iterative dialogue with ChatGPT, and refined their understanding under real-time faculty supervision. Faculty intervened only to correct conceptual inaccuracies, model evidence verification, and encourage metacognitive reflection.

### Assessment tools

Four blueprint-based MCQ forms (A-D) were developed, each with 20 vignette-based items targeting higher-order community medicine concepts. Items were reviewed by three senior faculty members, achieving a content validity index (CVI) of 0.92 and >90% inter-expert agreement. Pilot testing in 20 final-year MBBS students (not in the main study) confirmed psychometric equivalence. Items with difficulty indices between 0.30–0.80 and discrimination indices >0.20 were retained. Internal consistency was high (Cronbach’s alpha ≥ 0.80 for all forms). Student perceptions were assessed with a validated questionnaire, including a 5-point Likert scale (cognitive engagement, clarity, motivation, perceived educational value, satisfaction) and open-ended questions. The tool was faculty-reviewed, pre-tested, and administered anonymously via the LMS one month post-intervention.

### Facilitator training and fidelity monitoring

Facilitators completed a four-hour training programme on retrieval practice theory, Bloom’s Levels IV-VI, ethical AI use, and prompt engineering, delivered by a multidisciplinary team. Each received a standard operating procedures manual and fidelity checklist. Session logs and student reflections were reviewed weekly to ensure procedural adherence.

### Data management and ethics

Participants were assigned anonymised IDs, and data were stored on an encrypted server with role-based access control. The LMS enforced completion of mandatory fields, and 10% of entries were audited weekly. The analysis followed a per-protocol approach, including only participants who completed the baseline assessment (Form A) and attended at least three of the four intervention sessions. Students absent at baseline, attending fewer than three sessions, or withdrawing consent were excluded. Follow-up completion was maximised by supervised administration during scheduled sessions. For participants absent on the scheduled day of the one-month (Form C) or three-month (Form D) assessments, the research team met them individually within the following day to administer the test. This process achieved complete follow-up for all participants in the per-protocol cohort. Ethical approval was obtained prior to commencement of the study from the Institutional Human Ethics Committee for Faculty Research (CARE IHEC-II), Chettinad Academy of Research and Education (Deemed to be University), Tamil Nadu, India (Approval No: IHEC-II/1018/26; dated 23 January 2026). The study was conducted in accordance with the ethical principles outlined in the Declaration of Helsinki and in compliance with applicable national guidelines governing research involving human participants. Written informed consent was obtained from all participants prior to enrolment. Participation was voluntary, and no academic penalties were imposed for non-participation.

### Statistical analysis

Analyses were performed using SPSS v26 and R v4.3.0. Change across time (Forms A–D) was examined with repeated-measures ANOVA (reporting F, *p*, and partial η²), alongside a linear mixed-effects model with fixed effects for time, intervention sequence (early vs delayed), and their interaction, and random intercepts for cluster and participant. Model-based marginal means (±SE) and changes versus baseline with 95% confidence intervals were estimated. A sensitivity ANCOVA for the post-test (Form B), adjusting for baseline score and SES, was reported in the Supplement. Subgroup effects by baseline achievement category (lower-achieving vs higher-achieving students) were tested via the time × type interaction in the mixed model. Assumptions were checked (Shapiro–Wilk for normality; Mauchly’s test of sphericity with Greenhouse–Geisser correction when violated). Missingness was assessed with Little’s MCAR test; given low missingness, listwise deletion was used. Likert-scale items were summarised as means ± SD and five-category distributions; Cronbach’s *α* was computed for internal consistency. Qualitative data underwent reflexive thematic analysis (Braun & Clarke) by two independent coders (*κ* reported), with discrepancies resolved by consensus.

## Results

Of the 270 third-year MBBS students invited, 17 were excluded for not meeting the per-protocol definition—absence at the baseline assessment and/or attendance at fewer than three of the four intervention sessions—yielding a final analytic sample of 253 students. Specifically, 9 students were absent at baseline and 8 attended fewer than three sessions. All participants in the per-protocol cohort completed assessments at baseline, immediate post-test, one-month, and three-month follow-up. Of these, 121 were in the early-intervention sequence (clusters A and B) and 132 in the delayed-intervention sequence (clusters C and D). Baseline characteristics, including age, gender distribution, academic classification, baseline MCQ scores, socio-economic status, device used for LMS access, prior ChatGPT exposure, and residence status, were comparable between groups, with lower middle class being the most common socio-economic category. No statistically significant baseline differences were observed for any measured variable (Table 1). Results are presented according to two predefined grouping structures: (i) early versus delayed intervention sequences to assess timing effects, and (ii) baseline achievement categories to evaluate differential responsiveness to the intervention.

**Table 1.** Baseline Demographic, Academic, and Technological Characteristics of Third-Year MBBS Participants by Intervention Sequence in a Competency-Based Medical Education Setting (*n* = 253).

Variable	Early Intervention (A & B) (*n* = 121)	Delayed Intervention (C & D) (*n* = 132)	Total (*n* = 253)	*p*-value
**Age (years), mean ± SD**	22.11 ± 1.42	22.03 ± 1.32	22.07 ± 1.37	0.655
**Gender**				0.160
Male	73 (60.3%)	67 (50.8%)	140 (55.3%)	
Female	48 (39.7%)	65 (49.2%)	113 (44.7%)	
**Academic classification (Lower-achieving/Higher-achieving)**				0.376
Lower-achieving	70 (57.9%)	68 (51.5%)	138 (54.5%)	
Higher-achieving	51 (42.1%)	64 (48.5%)	115 (45.5%)	
**Baseline MCQ score (Form A), mean ± SD**	61.83 ± 4.27	61.72 ± 4.80	61.77 ± 4.55	0.851
**Socio-economic status (Modified Kuppuswamy)**				0.799
Lower Middle	80 (66.1%)	85 (64.4%)	165 (65.2%)	
Upper Lower	14 (11.6%)	19 (14.4%)	33 (13.0%)	
Upper Middle	27 (22.3%)	28 (21.2%)	55 (21.7%)	
**Primary device for LMS (Laptop/Phone)**				0.769
Laptop	50 (41.3%)	58 (43.9%)	108 (42.7%)	
Phone	71 (58.7%)	74 (56.1%)	145 (57.3%)	
**Prior ChatGPT exposure**				1.000
Yes	61 (50.4%)	67 (50.8%)	128 (50.6%)	
No	60 (49.6%)	65 (49.2%)	125 (49.4%)	
**Residence (Hosteller/Day scholar)**				0.164
Hosteller	60 (49.6%)	78 (59.1%)	138 (54.5%)	
Day scholar	61 (50.4%)	54 (40.9%)	115 (45.5%)	
**Usual mode of study (Textbook/Digital/Mixed)**				0.062
Textbook	85 (70.2%)	77 (58.3%)	162 (64.0%)	
Digital	36 (29.8%)	55 (41.7%)	91 (36.0%)	
Mixed	0 (0.0%)	0 (0.0%)	0 (0.0%)	

**Figure 1.**
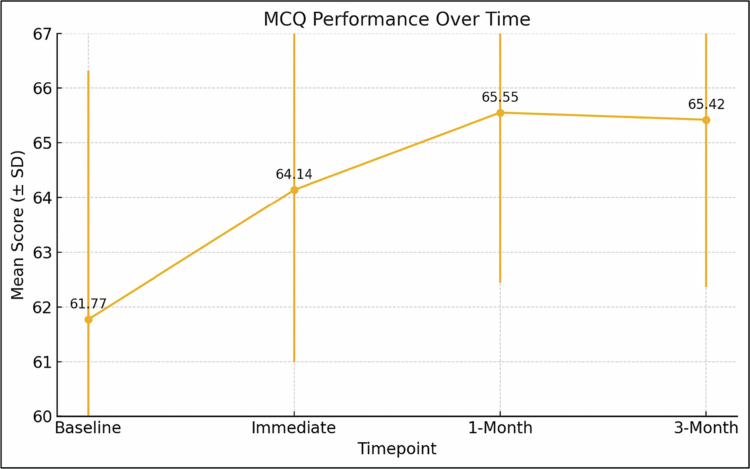
Change in Multiple-Choice Question (MCQ) Performance from Baseline to 3-Month Follow-up Showing Sustained Improvement Over Time (*n* = 253).

Students’ MCQ scores improved markedly over the study period. The repeated-measures ANOVA showed a significant overall effect of time (F [3, 756] = 65.14, *p* < 0.001), and the mixed-effects model confirmed gains at each follow-up compared with baseline. Immediately after the intervention, scores increased by an adjusted mean of 2.37 points (95% CI: 1.81–2.93; *p* < 0.001, Cohen’s d = 0.62). These improvements did not fade; scores at one month were 3.78 points higher than baseline (95% CI: 3.22–4.34; *p* < 0.001, d = 0.99) and at three months were 3.65 points higher (95% CI: 3.09–4.21; *p* < 0.001, d = 0.95) (Table 2; Figure 1).

**Table 2.** Adjusted Estimates of Multiple-Choice Question (MCQ) Performance Over Time Using Linear Mixed-Effects Model and Repeated-Measures ANOVA (*n* = 253).

Timepoint	Raw Mean ± SD	LMM Adj. Mean ± SE	Adj. Δ vs Baseline	95% CI for Δ	*p* (LMM)	Cohen’s d	RM-ANOVA F(df), p
Baseline (Form A)	61.77 ± 4.55	61.77 ± 0.29	−	−	−	−	65.14 (3, 756), *p* < 0.001
Immediate Post-test (Form B)	64.14 ± 3.15	64.14 ± 0.29	2.37	1.81 to 2.93	<0.001	0.62	
1-Month Follow-up (Form C)	65.55 ± 3.11	65.55 ± 0.29	3.78	3.22 to 4.34	<0.001	0.99	
3-Month Follow-up (Form D)	65.42 ± 3.06	65.42 ± 0.29	3.65	3.09 to 4.21	<0.001	0.95	

**Figure 2.**
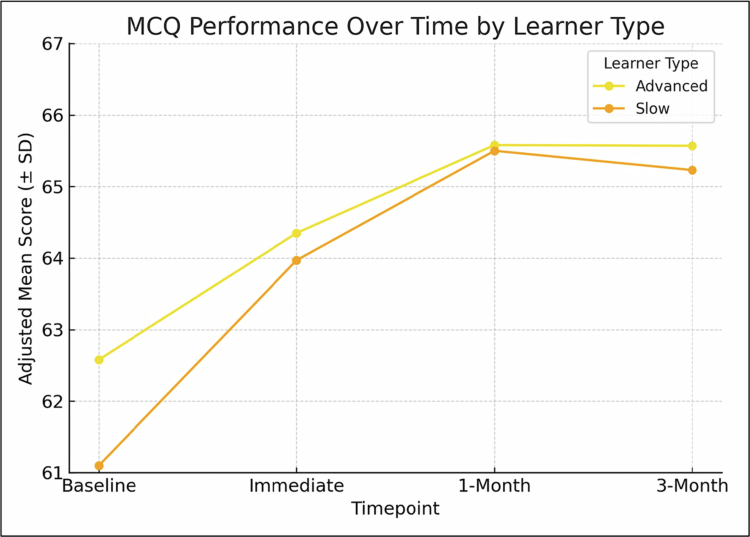
Adjusted Mean Higher-Order MCQ Scores Over Time by Learner Type (*n* = 253).

Both Lower-achieving and Higher-achieving students benefited from the intervention, but the effect was more pronounced in the Lower-achieving-learner group. Immediately after the intervention, Lower-achieving students improved by 2.87 points (95% CI: 1.90–3.84; *p* < 0.001, d = 0.72), whereas Higher-achieving students gained 1.77 points (95% CI: 0.80–2.74; *p* < 0.001, d = 0.49). The difference in improvement between the two groups was not statistically significant when tested as a time × learner type interaction, and both maintained their gains at one and three months (Table 3; Figure 2).

**Table 3.** Subgroup Effects of the Intervention on Multiple-Choice Question (MCQ) Performance by Learner Type Using Linear Mixed-Effects Model (*n* = 253).

Learner Type	Adj. Δ A→B	95% CI for Δ	Cohen’s d	*p* (LMM)	1-M Adj. Mean ± SE	3-M Adj. Mean ± SE	Time × Type p
Lower-achieving students	2.87	1.90 to 3.84	0.72	<0.001	65.59 ± 0.50	65.57 ± 0.50	0.15
Higher-achieving students	1.77	0.80 to 2.74	0.49	<0.001	65.50 ± 0.50	65.23 ± 0.50	0.15

Feedback on the ChatGPT-assisted sessions was overwhelmingly positive. Overall satisfaction received the highest average score (4.25 ± 0.88), closely followed by cognitive engagement (4.15 ± 0.92). Motivation and perceived educational value also scored well. The only relatively lower rating came for clarity of interaction (3.39 ± 1.19), suggesting that the flow and precision of AI–student exchanges could be improved (Table 4).

**Table 4.** Student Perception of ChatGPT-Assisted Learning Sessions Across Five Domains (*n* = 253).

Domain	Mean ± SD	Strongly Agree *n* (%)	Agree *n* (%)	Neutral *n* (%)	Disagree *n* (%)	Strongly Disagree *n* (%)
Cognitive engagement	4.15 ± 0.92	107 (42.3%)	93 (36.8%)	40 (15.8%)	9 (3.6%)	4 (1.6%)
Clarity of interaction	3.39 ± 1.19	56 (22.1%)	62 (24.5%)	76 (30.0%)	42 (16.6%)	17 (6.7%)
Motivation	4.15 ± 1.03	114 (45.1%)	95 (37.5%)	22 (8.7%)	12 (4.7%)	10 (4.0%)
Educational value	3.90 ± 0.89	132 (52.2%)	79 (31.2%)	33 (13.0%)	5 (2.0%)	4 (1.6%)
Overall satisfaction	4.25 ± 0.88	122 (48.2%)	85 (33.6%)	35 (13.8%)	9 (3.6%)	2 (0.8%)

Analysis of students’ written reflections revealed three recurring themes. The first, and most common, was AI as a cognitive scaffold (71.5%), with students emphasising how ChatGPT’s feedback helped them correct errors and refine their reasoning in real time. The second theme, improved self-awareness (55.7%), reflected the role of the sessions in prompting metacognitive reflection and boosting confidence. A smaller but notable proportion (25.5%) raised concerns over over-reliance, voicing fears about reduced independent thinking or exposure to AI-generated inaccuracies (Table 5).

**Table 5.** Thematic Analysis of Student Reflections (*n* = 235).

Theme	Sub-theme/Code(s)	Illustrative Quotes (≤30 words)	Prevalence *n* (%)	Valence
AI as cognitive scaffold	Real-time feedback; Concept clarification	‘ChatGPT pinpointed my flawed logic and guided me to the correct differential—like having a tutor on demand.’ ‘It helped me connect concepts I usually study in isolation.’	168 (71.5)	+
Improved self-awareness	Metacognitive reflection; Confidence calibration	‘Reflecting after each session showed my growth curve and boosted my confidence in tackling clinical scenarios.’ ‘I realised I often jump to answers without considering alternatives.’	131 (55.7)	+
Concerns over over-reliance	Authentic-thinking erosion; Hallucination fear	‘I fear I might lean too much on ChatGPT and skip the productive struggle that cements learning.’ ‘Sometimes the AI gave convincing but wrong answers—I need to double-check facts.’	60 (25.5)	–

Note: Prevalence (%) calculated as the number of students mentioning the theme divided by the total number providing reflections (*n* = 235).

Adherence to the intervention protocol was high. All facilitators completed training, prompt usage consistency exceeded 94%, and both reflection submission and session log completion rates were above 92% (Supplementary Table S1). These fidelity metrics align with the implementation component of the hybrid Type II framework, supporting the feasibility and reproducibility of the intervention in real-world educational settings. In the sensitivity analysis, after adjusting for baseline scores and socio-economic status, there was no significant difference in post-test performance between early- and delayed-intervention sequences (adjusted mean difference 0.15; 95% CI: –0.63 to 0.93; *p* = 0.703), indicating that the timing of the intervention did not influence the outcomes (Supplementary Table S2).

## Discussion

Our study demonstrated that integrating ChatGPT into a structured, faculty-supervised retrieval practice framework led to significant gains in higher-order MCQ performance among third-year MBBS students, with improvements evident immediately after the intervention and sustained at one- and three-month follow-ups. These benefits were observed across both Lower-achieving and Higher-achieving students, though the effect size was more pronounced in the Lower-achieving-learner group. This suggests that AI-assisted scaffolding may offer particular value for those requiring additional support. Student feedback reflected high satisfaction, strong cognitive engagement, and increased motivation, though some participants noted challenges with the clarity and flow of AI–student interactions. To our knowledge, this is among the first studies in India’s CBME context—and one of the few globally—to combine supervised use of a generative AI tool with retrieval practice explicitly targeting Bloom’s higher-order cognitive domains (Levels IV–VI), providing early evidence for its feasibility and educational impact.

The comparatively lower rating for ‘clarity of AI interaction’ (mean 3.39 ± 1.19) suggests that, while students valued the sessions, some encountered challenges in interpreting ChatGPT’s responses. Qualitative reflections indicated that certain outputs were verbose, variably structured, or employed unfamiliar terminology inconsistent with textbook phrasing, leading to temporary confusion [6,7]. Others reported difficulty in constructing effective follow-up prompts, occasionally resulting in circular exchanges or minor factual inconsistencies. These experiences reflect early cognitive-interface limitations described in recent educational AI research, where response clarity depends strongly on user prompting skill and contextual specificity. To enhance future clarity, brief pre-session AI-literacy training and exemplar prompt templates could be introduced, alongside faculty modelling of concise, evidence-oriented follow-up questioning [8]. Such refinements would improve the interpretive precision of AI–student dialogue while preserving the high cognitive demand central to retrieval-based learning [9].

Although this study demonstrated sustained improvements in higher-order MCQ performance, it is important to acknowledge that even blueprint-based, vignette-driven MCQs can only approximate the full range of competencies potentially shaped by generative-AI–assisted learning. Beyond cognitive recall and analytical reasoning, tools such as ChatGPT may influence more complex domains like diagnostic reasoning, adaptive problem-solving, and collaborative decision-making that are less amenable to quantification through standard MCQ formats. Future investigations should therefore employ mixed-method and performance-based assessments, including OSCE-style tasks, reflective writing, and portfolio analyses, to capture the richer cognitive and metacognitive effects of AI scaffolding. Similar recommendations have been advanced in recent systematic reviews of generative AI in medical education, underscoring the need for multimodal evaluation frameworks that align assessment methods with the multidimensional nature of higher-order learning [10].

The testing (retrieval) effect reliably strengthens long-term retention, transfer, and understanding in health-professions education. Repeated, effortful recall with feedback outperforms restudy and helps preserve knowledge over time [11]. These benefits are not confined to any single curriculum model but have been demonstrated in diverse contexts, from US medical schools to UK postgraduate training and even in non-medical domains such as engineering, law, and psychology education—where retrieval plus structured feedback has consistently improved problem-solving and applied reasoning skills [12–14]. Our one- and three-month gains are therefore theoretically expected and likely amplified by structured prompts that required justification, surfaced misconceptions, and guided refinement. This extends a literature historically centred on recall to show that, when paired with scaffolded feedback, retrieval can also support higher-order competencies—analysis, synthesis, and evaluation—central to both India’s CBME and international competency frameworks such as the UK General Medical Council (GMC) *Outcomes for Graduates* [15], the AAMC *Core Entrustable Professional Activities* [16], and the CanMEDS Scholar Role [17].

In medical education, supervised ChatGPT can do more than simplify content. Within a guided workflow, it can generate exam-style questions, elicit stepwise reasoning, and deliver rapid, targeted explanations—affordances documented in practice-oriented literature [18]. These benefits, however, are tempered by cautions about factual error and over-reliance [19]. This aligns with recent international policy guidance from UNESCO, which calls for human-centred, transparent, and supervised integration of generative AI in education [20]. Globally, health-professions regulators are adopting similar positions: the GMC in the UK and the AAMC in the US both emphasise digital professionalism and critical appraisal of AI outputs as part of graduate outcomes. Meta-analytic evidence across higher education now indicates a moderate positive effect of ChatGPT on academic performance and higher-order thinking, with more mixed effects on perceptions [21]. Our longitudinal design adds the important dimension of durability in a real-world CBME context.

The larger gains among Lower-achieving students are consistent with cognitive load reduction under guidance: structured prompts decompose complex tasks, immediate AI feedback targets misconceptions at the point of need, and brief reflections consolidate learning. Guided GenAI has been associated with deeper knowledge development, creativity, and engagement, while unguided use risks inaccuracy and shallow processing [22]. This principle is universally relevant—benefiting students in Indian MBBS programmes, students in resource-rich environments where AI access is ubiquitous, and students in low-resource settings where faculty contact time is limited. Indeed, parallel findings in engineering and psychology suggest that guided AI-assisted retrieval benefits underperforming students disproportionately, making it a potential equity lever across disciplines [23]. While the delayed-intervention, quasi-experimental design used in this study allowed all participants eventual exposure and controlled for inequity, it cannot fully eliminate residual confounding or institutional-context effects. Future research should therefore include multi-institutional, randomised controlled trials to validate these findings under diverse curricular, linguistic, and technological conditions. Such trials could quantify both efficacy and implementation fidelity across settings, identify moderators of effect (e.g., baseline digital literacy, faculty training quality), and determine the sustainability of learning gains once generative-AI use becomes routine within CBME curricula.

Because generative AI unsettles legacy testing formats, converging evidence and expert consensus recommend redesigning assessment to assume AI use and emphasise authentic, higher-order tasks requiring application, analysis, and synthesis [24] the same cognitive profile targeted by our vignette-based retrieval activities with written justifications. This is consistent with WFME global standards [25], which encourage assessment strategies that measure reasoning and professional judgement over rote recall. The relatively lower ‘clarity’ ratings in our study point to a practical need for prompt templates and short AI-literacy sessions to optimise AI–student exchanges without diluting cognitive demand. These adjustments align with broader recommendations for human-centred, supervised AI integration in education [26] and with recent proposals from the US National Board of Medical Examiners and UK medical schools for integrating AI-awareness into formative and summative assessments [27]. By embedding these strategies within global competency frameworks, our model offers a transferable blueprint for medical educators worldwide—whether operating under India’s CBME mandates, the GMC’s graduate outcomes, the AAMC’s EPAs, or WFME standards. This ensures that supervised, retrieval-based AI integration is not an isolated innovation but a globally relevant, standards-aligned educational practice [28].

### Strength and limitations

This study has several notable strengths. First, it achieved complete enumeration of all eligible students with a prespecified per-protocol cohort and 100% follow-up—an uncommon level of retention in educational research—through supervised administration and next-day catch-ups. Second, high implementation fidelity was maintained across facilitators, prompts, reflections, and logs, supported by structured faculty training and an auditable protocol. Third, the faculty-supervised design provided a controlled and transparent way to integrate ChatGPT into retrieval practice, mitigating factual inaccuracies while fostering metacognitive reflection. Fourth, longitudinal follow-up at one and three months confirmed the durability of higher-order learning gains, with particularly marked benefits among Lower-achieving students, highlighting the equity potential of guided AI interventions. Finally, by embedding the intervention within a Hybrid Type II framework, the study evaluated both effectiveness and real-world feasibility, offering a scalable, timetable-compatible model for CBME settings. Limitations include the non-randomised, phased rollout, which cannot fully eliminate residual confounding, and the single-institution setting in South India, which may limit generalisability to other medical schools or different cultural and curricular contexts. The primary outcome relied solely on higher-order MCQs that, despite blueprinting and validation, assess mainly convergent reasoning rather than the open-ended, collaborative, and creative problem-solving potentially fostered by AI-mediated learning. Future studies should complement MCQs with performance-based and reflective assessments (e.g., OSCEs, portfolios) to provide a fuller evaluation. The per-protocol analysis may slightly overestimate efficacy, while lower clarity ratings highlight the need for standardised prompt templates and brief AI-literacy orientation. Finally, a novelty effect cannot be excluded; students’ heightened engagement and performance gains may reflect the initial appeal of AI-assisted learning and should be reassessed once such interventions become routine in medical curricula.

## Conclusion

In a real-world CBME environment, a supervised, retrieval-anchored use of ChatGPT led to sustained improvements in higher-order learning, with disproportionate benefits for students needing greater cognitive scaffolding. By repositioning generative AI from a passive information source to an active cognitive partner, the intervention aligns with NMC competencies and international standards such as the GMC Outcomes for Graduates, AAMC Core EPAs, CanMEDS Scholar Role, and WFME global benchmarks. Importantly, this model is cost-sensitive (USD 4.50 per student for the full study period), minimally burdensome once prompt templates and SOPs are established, and adaptable across diverse institutional contexts, including those with limited resources. Medical educators worldwide should consider integrating supervised AI into competency-based curricula, coupled with structured prompt-engineering support, metacognitive reflection, and AI-literacy orientation, to enhance learning while safeguarding independent reasoning. With thoughtful governance, fidelity monitoring, and alignment to recognised competency frameworks, retrieval-based AI integration should progress from an innovative pilot to a standardised CBME teaching strategy in medical schools globally. Future research should include multi-institutional, cluster-randomised trials that compare retrieval + AI against retrieval-only and AI-only conditions, extend outcomes to performance-based assessments, and explore long-term sustainability beyond any initial novelty effects.

## Data Availability

Data will be shared upon reasonable request to the corresponding author for academic purpose only.
